# Vγ9Vδ2 T Cells Concurrently Kill Cancer Cells and Cross-Present Tumor Antigens

**DOI:** 10.3389/fimmu.2021.645131

**Published:** 2021-06-02

**Authors:** Gitte Holmen Olofsson, Manja Idorn, Ana Micaela Carnaz Simões, Pia Aehnlich, Signe Koggersbøl Skadborg, Elfriede Noessner, Reno Debets, Bernhard Moser, Özcan Met, Per thor Straten

**Affiliations:** ^1^ National Center for Cancer Immune Therapy, CCIT-DK, Department of Oncology, Copenhagen University Hospital Herlev, Herlev, Denmark; ^2^ Department of Biomedicine, Aarhus University, Aarhus, Denmark; ^3^ Helmholtz Zentrum München, Germany Research Center for Environmental Health, Immunoanalytics, Research Group Tissue control of immunocytes, Munich, Germany; ^4^ Laboratory of Tumor Immunology, Department of Medical Oncology, Erasmus MC-Cancer Center, Rotterdam, Netherlands; ^5^ Division of Infection & Immunity, Cardiff University School of Medicine, Cardiff, United Kingdom; ^6^ Department of Immunology and Microbiology, Faculty of Health and Medical Sciences, University of Copenhagen, Copenhagen, Denmark

**Keywords:** γδ or gamma delta T cells, Vγ9Vδ2 T cells, APC or antigen presenting cells, antigen cross-presentation, cancer, cancer killing

## Abstract

The human Vγ9Vδ2 T cell is a unique cell type that holds great potential in immunotherapy of cancer. In particular, the therapeutic potential of this cell type in adoptive cell therapy (ACT) has gained interest. In this regard optimization of *in vitro* expansion methods and functional characterization is desirable. We show that Vγ9Vδ2 T cells, expanded *in vitro* with zoledronic acid (Zometa or ZOL) and Interleukin-2 (IL-2), are efficient cancer cell killers with a trend towards increased killing efficacy after prolonged expansion time. Thus, Vγ9Vδ2 T cells expanded for 25 days *in vitro* killed prostate cancer cells more efficiently than Vγ9Vδ2 T cells expanded for 9 days. These data are supported by phenotype characteristics, showing increased expression of CD56 and NKG2D over time, reaching above 90% positive cells after 25 days of expansion. At the early stage of expansion, we demonstrate that Vγ9Vδ2 T cells are capable of cross-presenting tumor antigens. In this regard, our data show that Vγ9Vδ2 T cells can take up tumor-associated antigens (TAA) gp100, MART-1 and MAGE-A3 - either as long peptide or recombinant protein – and then present TAA-derived peptides on the cell surface in the context of HLA class I molecules, demonstrated by their recognition as targets by peptide-specific CD8 T cells. Importantly, we show that cross-presentation is impaired by the proteasome inhibitor lactacystin. In conclusion, our data indicate that Vγ9Vδ2 T cells are broadly tumor-specific killers with the additional ability to cross-present MHC class I-restricted peptides, thereby inducing or supporting tumor-specific αβTCR CD8 T cell responses. The dual functionality is dynamic during *in vitro* expansion, yet, both functions are of interest to explore in ACT for cancer therapy.

## Introduction

Conventional T cells expressing αβ T cell receptors (TCR) have been characterized in detail with regards to antigen recognition, differentiation, and function (1). γδ T cells are less well characterized, less abundant, and exist as several subtypes with the common feature that they express a dimeric TCR consisting of a γ- and a δ-chain. The dominant γδ T cell subtype in peripheral blood are Vγ9Vδ2 T cells which are only found in humans, higher primates and the alpaca (2), and constitute 0.5-10% of lymphocytes in human blood (3). Most Vγ9Vδ2 T cells are double negative (DN) for the co-receptors CD4 and CD8, approx. 20-30% are single positive CD8 and 0.1-7% express CD4 (4, 5). The functional role of these co-receptors in the context of γδ T cells is however unknown, since Vγ9Vδ2 T cells recognize antigen in an HLA independent fashion. To this end, Vγ9Vδ2 T cells recognize a group of non-peptide antigens called phosphoantigens (pAgs) (6), examples of these are the bacterial metabolite ((E)-4-hydroxy-3-methyl-but-2-enylpyrophosphate (HMBPP)) (7) and isopentenyl pyrophosphate (IPP), which is a by-product of the mevalonate isoprenoid pathway. The interaction between pAgs and the butyrophilin proteins BTN3A1 (8, 9) and BTN3A2 (10, 11) leads to extracellular changes in conformation (12), allowing for proper recognition of the Vγ9Vδ2 TCR (13). Normal or healthy cells have low levels of IPP, which does not activate Vγ9Vδ2 T cells. In contrast, stressed cells and cancer cells show increased IPP levels, although in most cases not enough in itself for recognition by Vγ9Vδ2 T cells (14, 15). However, the activation of Vγ9Vδ2 T cells by pAgs can be exploited using drugs such as zoledronic acids (ZOL) (16). ZOL is an aminobisphosphonate that inhibits the enzyme farnesyl pyrophosphate synthase in the mevalonate pathway, which induces an accumulation of IPP in the cell (17, 18). ZOL can be used for selective expansion of Vγ9Vδ2 T cells from blood samples, and also to sensitize cancer cells to Vγ9Vδ2 T cell-mediated killing. Thus, the addition of ZOL to cultures of PBMC along with interleukin-2 (IL-2) leads to a selective expansion of Vγ9Vδ2 T cells, which are in turn highly efficacious killers of cancer cells upon sensitization of cancer cells with ZOL. Induction of effector function is not solely governed by recognition of pAgs, but also influenced by expression of receptors traditionally attributed to NK cells, such as NKG2D and DNAM-1 (3, 19).

An additional characteristic of Vγ9Vδ2 T cells is the capacity to cross-present antigen, i.e., to act as antigen presenting cells (APCs) (20). The term APC is normally used to refer to a group of innate cells that mediate cellular immune responses by processing and presenting antigens to aβ T cells. Classical APCs include dendritic cells (DCs) and macrophages, but Vγ9Vδ2 T cells have also been shown to cross-present viral and tumor antigen (21). Cross-presentation of the melanoma-associated antigen MART-1 was demonstrated using long synthetic peptide (22), and uptake of cellular protein upon killing of cancer cells has also been reported (23). However, in the latter case, efficient cross-presentation only took place when cancer cells were opsonized (24), and involvement of the proteasome was not investigated. Although some antigens have been shown to be cross-presented independently of proteasomal degradation, in most cases the proteasome is crucial for cross-presentation (25).

In the past decade, immunotherapy has revolutionized treatment of cancer and given new hope to patients with metastatic disease (26). Adoptive cell therapy (ACT) with tumor infiltrating lymphocytes (TILs) or T cells equipped with chimeric antigen receptors (CARs) have yielded impressive results in melanoma, and hematological malignancies, respectively (27). To the former, administration of *in vitro* expanded TILs is associated with 50% objective and 20% complete responses (28). Concerning ACT using CARs, administration of CAR T cells recognizing CD19 are now approved for the treatment of acute lymphoblastic leukemia (ALL) and diffuse large B-cell lymphoma (DLBCL) (29). In particular, CAR therapy is a highly promising broadly applicable strategy with the potential to develop patient tailored, off the shelf treatments. Great advances have been made over the past few years (30), but much need to be learned, in particular, in terms of optimal targets and best suited cell types for ACT.

The majority of studies on ACT, including FDA/EMA approved CAR therapies (31), are based on the use of conventional αβ-T cells as effector cells, largely, because these are well-studied effector cells in natural anti-cancer immunity with proven success in treatment settings (30). NK cells and γδ T cells have been tested as well in ACT treatments, with demonstrable pros and cons. NK and γδ T cells are capable of killing cancer cells in an HLA unrestricted manner, with the potential of efficacy in the absence of graft-versus-host disease (GvHD), and can be used as an off-the-shelf cellular source even in an allogenic setting (32). NK cells are problematic in terms of expansion of primary cells, conversely, Vγ9Vδ2 T cells are easily expanded to high cell numbers using ZOL and IL-2. Several clinical trials based on administration of *in vitro* expanded Vγ9Vδ2 T cells have been carried out with encouraging data, including good tolerability and little or no toxicity. But studies included too few patients to draw conclusions on clinical response (33). The combined capacity to kill cancer cells and cross-present antigen to CD8 T cells – even when equipped with a CAR (22) - represents another feature in favor of future testing of *in vitro* expanded Vγ9Vδ2 T cells in ACT in cancer. We describe that Vγ9Vδ2 T cells expanded with ZOL and IL-2 are capable of killing cancer cells as well as cross-presenting tumor antigens. Moreover, we show the dynamic change of this dual functionality over time in culture, a characteristic that should be considered in clinical application.

## Materials and Methods

### Samples From Patients and Healthy Donors

Peripheral blood mononuclear cells (PBMC) from healthy donors were obtained from the blood bank at Rigshospitalet, Copenhagen, Denmark. Processing was completed within < 6 h for all sample specimens. PBMC were isolated by centrifugation with Lymphoprep™ (Axis-Shield PoC) (30 minutes at 1200 RPM) and cryopreserved at -150°C in fetal bovine serum (FBS) (GibcoBRL) + 10% dimethylsulfoxide (DMSO) (Sigma-Aldrich) using a CoolCell^®^ (Bioscision) gradual freezing device. Cells were thawed in pre-warmed 37°C RPMI and counted after thawing using trypan blue staining and a microscope.

### Cancer Cell Cultures

Cancer cell lines A2058 (melanoma), MDA-MB-231 (breast cancer), PC-3 (prostate cancer), U266 (myeloma) and K562 (chronic myelogenous leukemia) were all purchased from the American Type Culture Collection (ATCC). The FM55-1 (melanoma, ESTDAB-012) and FM86 (melanoma, ESTDAB-028) cancer cell lines were obtained from European Searchable Tumor Cell Line and Data Bank (ESTDAB) (http://www.ebi.ac.uk/ipd/estdab/). All cancer cells were grown in RPMI 1640 GlutaMAX-I™ medium (RPMI, Gibco) supplemented with 10% FBS (R10). Prior to cytotoxicity assays, the cancer cells were left untreated or pre-treated with 10 µM ZOL for 24 h.

### Expansion of Vγ9Vδ2 T Cells

Vγ9Vδ2 T cells were cultured in X-vivo 15 medium (Lonza) supplemented with 5% human serum (X-vivo +5% HS) (Sigma-Aldrich). Vγ9Vδ2 T cells were expanded from thawed PBMC. On day zero, 1x10^6^ PBMC were cultured in a 24 well plate with 2 ml X-vivo + 5% HS and stimulated with 10 µM zoledronic acid (ZOL, Zometa 4 mg/5ml, Novartis). On day 2, 1000 U/ml IL-2 (Preprotech) was added, and every second or third day onwards, the cultures were supplemented with 1 ml fresh medium and 1000 U/ml IL-2. Purity of the Vγ9Vδ2 T cells was tested at day 9 by flow cytometry. We are aware that different groups use different amount of ZOL and IL-2. In our hands, we found expansion of Vγ9Vδ2 T cells to be most efficient when using 10µM ZOL, combined with either high IL-2 (1000U/ml) or low IL-2 + low IL-15 (100U/ml IL-2 + 100U/ml IL-15) (34). We have also tested 1µM ZOL combined with 100U/ml IL-2, but our data gave a less efficient and less pure expansion of Vγ9Vδ2 T cells (data not published).

### Establishment of CMV Peptide Specific T Cell Cultures

αβTCR T-cell culture specific for CMV (CMV short) was generated from healthy donor (HD220) by stimulating with irradiated CMV-peptide-loaded Vγ9Vδ2 T cells (as an alternative to DCs, which are used in other protocols [32]). The following day, 40 U/ml IL-7 and 20 U/ml IL-12 (PeproTech) were added. Stimulation of the cultures was carried out every 8 days with CMV-peptide–loaded irradiated autologous Vγ9Vδ2 T cells. The day after peptide stimulation, 120 U/ml IL-2 (PeproTech) was added. Specificity of the T cell culture was tested by chromium release assays and IFNγ ELISPOT.

### mRNA Transfection of PBMC for the Generation of gp100 Specific αβTCR T Cells

T cells expressing αβTCR specific for gp100 (gp100 short) were generated by mRNA transfection. The coding sequence of the gp100_280-288_-specific TCR 296 (35) α and β chain was *de novo* synthesized and cloned into the pCIpA_102_ plasmid (kindly provided Dr. G. Gaudernack, The Norwegian Radium Hospital, Oslo, Norway). Plasmids were linearized, purified (Wizard DNA Clean-Up System (Promega, Oslo, Norway), and *in vitro* transcribed 36, and used for electroporation of PBMC from healthy donors. PBMC were washed twice in OptiMEM medium (Invitrogen) and adjusted to a final cell density of 1 x 10^8^ cells/ml in the same media. The cell suspension was pre-incubated for 5 min on ice and gp100 TCR encoding mRNA (100 µg/ml final concentration) was added to PBMCs before transfer to a 4-mm gap electroporation cuvette. Cells were pulsed using a BTX 830 square-wave electroporator (Harvard Apparatus, Holliston MA, USA), adjusted to a single pulse, 500 V, 5 ms. After electroporation, cells were transferred to pre-warmed culture medium and incubated in humidified atmosphere with 5% CO_2_. Mock-(H_2_O) transfected PBMCs were used as controls. Specificity of the T cell culture was tested by chromium release assays and IFNγ ELISPOT.

### Retroviral Transduction for the Generation of MART-1 Specific T Cells

TCRα and β sequences of the HLA-A2-restricted MART-1-specific A42 T cell clone (37) were codon optimized and murinized by exchanging the constant regions by their murine counterparts then linked by a P2A element to yield the transgene cassette 5’-TCRvß4.2J2.7-P2A-TCRvα29J42.01-3’ (IMGT nomenclature). The cassette was cloned into the retroviral vector MP71-PRE (38) using NotI and EcoRI restriction sites. This vector was designated MP71-TCR-A42.

Transgenic TCR expression in T cells was achieved as described (38). Briefly, PBMCs were plated into 24-well plates at a cell density of 1 x 10^6^/ml per well in RPMI1640 supplemented with 10% human serum, 1% L-glutamine, 1% non-essential amino acids, 1% sodium pyruvate, 1% penicillin/streptomycin (all Invitrogen) and 100 U/ml IL-2 (Cancernova). Then, cells were activated with 5 μg/ml OKT3 (provided by E. Kremmer, Helmholtz Center Munich, Germany) and 1 μg/ml anti-CD28 (BD Pharmingen) for 2 days.

Amphotrophic TCR-A42-encoding retroviruses were generated as described (39) using TransIT^®^-LT1 Reagent (Mirus) according to the manufacturer’s protocol. Virus supernatant was harvested after 48 h and bound to RetroNectin^®^ (10 μg/ml, Takara) coated plates by centrifugation.

PBMCs, which were activated for 2 days, were added to virus-coated plates for 24 h, then split to freshly virus-coated plates and cultivated for another 3 days. Transduced PBMCs were transferred to uncoated plates and cultivated for at least 12 additional days, reducing the amount of IL-2 to 50 U/ml. TCR-A42 surface expression was determined at day 12 after transduction using anti-mouse TCRß-constant region-Pacific Blue (BioLegend) and anti-human CD8α-V500 (BD Pharmingen) antibodies. Furthermore, specificity of the T cell culture was also tested by chromium release assays and IFNγ ELISPOT.

### Lentiviral Transduction for the Generation of MAGE-A3 Specific T Cells

Lentiviral vector containing the high affinity MAGE-A3^a3a^ TCR (40) and corresponding packaging and envelope plasmids (VSVG, REV and gag/pol) was generously provided by Dr Andrew Gerry and Dr. Bent Jakobsen, Adaptimmune, Ltd. (Oxfordshire, UK) (41).

Lentivirus were produced and T cells transduced as previously described (42). In brief, 293T human embryonic kidney cells cultured in DMEM (BioWhittaker, Rockville MD, USA), 10% FBS, were transfected using 1 µg of pMAGE-A3^a3a^ TCR and 0.5 µg of corresponding packaging and envelope plasmids, together with TurboFect Transfection Reagent (Thermo Fisher Scientific). After 48 h, lentiviral supernatant was harvested. T cells were transduced by incubation with filtered lentiviral supernatant + 1000 U/ml rhIL-2 for 72 h before sorting using a FACSAria cell sorter (BD Biosciences, San Jose CA, USA).

1-2 x 10^5^ of the transduced and sorted T cells were put into a rapid expansion protocol (REP) consisting of feeder cells (a mix of three HD PBMCs, which were gamma irradiated with 40 Gy) at a ratio of 1:200 in T25 tissue culture flasks (Corning, #430168), 20 ml x-vivo medium + 5% human serum, 30 ng/ml anti-CD3 (OKT-3, eBioscience, #14-0037-82) and 6000 U/ml rhIL-2. On day 14 of REP, T cells were harvested and routine assays for receptor expression *via* flow cytometry and mycoplasma were conducted. Furthermore, specificity of the T cell culture was tested by chromium release assays and IFNγ ELISPOT.

### Flow Cytometry

Flow cytometry was used to study expression of surface- and intracellular markers on cells. In short, cells were washed twice in FACS buffer (PBS +2%FCS) before and after staining. Antibodies were mixed to a total volume of 50 µl, and cells were stained for 30 minutes at 4°C. **Table 1**, provides an overview of antibodies used in this paper. Near-Infrared fixable dead cell stain from Invitrogen (Carlsbad, California, USA). The gating analysis was either performed with BD FACSDiva™ software or NovoExpress^®^ software from ACEA biosciences.

**Table 1:** List of antibodies used in this study.

Antibody	Fluorochrome	Clone	Company
CCR7	PE-Cy7	3D12	BD bioscience, New Jersey, USA
CD3	PE-Cy7	UCTH1	BD bioscience, New Jersey, USA
CD3	BV421	UCTH1	Biolegend, San Diego, California, USA
CD16	FITC	3G8	Biolegend, San Diego, California, USA
CD56	FITC	L307.4	Biolegend, San Diego, California, USA
CD56	PE	NCAM16.2	BD bioscience, New Jersey, USA
CD86	APC	IT2.2	Biolegend, San Diego, California, USA
CD161	BV421	DX12	BD bioscience, New Jersey, USA
DNAM	PerCP-Cy5.5	11A8	Biolegend, San Diego, California, USA
GPR56	PE	4C3	Biolegend, San Diego, California, USA
HLA-ABC	BV711	G46-2.6	BD bioscience, New Jersey, USA
HLA-DR	HV500	G46-6	BD bioscience, New Jersey, USA
IFNγ	BV510	4S.B3	Biolegend, San Diego, California, USA
IL-2	PE	MQ1-17H12	BD bioscience, New Jersey, USA
NKG2D	BV510	1D11	Biolegend, San Diego, California, USA
TNFα	PE-CF594	Mab11	BD bioscience, New Jersey, USA
TCRγ/δ	FITC	11F2	BD bioscience, New Jersey, USA
Vγ9	PC5	IMMU360	Beckmann Coulter, Brea, California, USA

### Intracellular Staining (ICS)

Vγ9Vδ2 cells were co-cultured with PC3 cells (cancer cells), either non-stimulated or stimulated with 10 µM ZOL, at a ratio (1:1). The cells were incubated for 5 hours in the presence of Brefeldin A (BioLegend). Addition of cell culture medium served as a negative control, while 5 ng/ml PMA (Sigma Aldrich) plus 75 nM Ionomycin (Sigma Aldrich) were used as a positive control. After incubation, cells were centrifuged and washed twice with PBS + 2% FBS. Staining with surface antibodies was performed as described above. Then cells were fixed and permeabilized as described in detail in the manual of the Intracellular Fixation & Permeabilization Buffer Set (eBioscience). In short, cells were fixed overnight at 4°C in 200 µl Fixation Buffer per well. After centrifugation, cells were washed twice with 150 µl Permeabilization Buffer per well. Staining with intracellular antibodies was then performed in the same manner as the surface antibody staining. Subsequently, cells were washed twice with Permeabilization buffer, resuspended in 150 µl PBS + 2% FBS and acquired on the NovoCyte Quanteon.

### Cell Sorting

Sorting was used to purify αβTCR-specific cultures and was performed on the FACS Aria(BD bioscience). For sorting, the MAGE-A3 transduced T cell cultures were first stained with HLA-A1 tetramers for 30 minutes at 37°C. For that, 20 ng of PE- and APC-conjugated MAGE-A3 specific tetramers were added to1 x 10^6^ T cells in 50 mL of 1x PBS, 0.5% bovine serum albumin (BSA, Sigma Aldrich) and 2 mM EDTA. After the tetramer staining, CD3-BV421 (Biolegend), in a volume of 50 µl, was added directly into the tetramer/cell mix and incubated for 20–30 minutes in the dark and on ice. The transduced MAGE-A3 (also named MAGE-A3a3a) tetramer positive T cells were sorted directly into a rapid expansion protocol (REP) for further culturing. All antibodies, buffers and procedures were kept under sterile conditions to ensure aseptic sorting of cells for further culturing.

### Rapid Expansion Protocol (REP)

To expand the MAGE-A3 positive T cells cells into high cells number, the rapid expansion protocol (REP) was used. For this, a REP mix was generated which included: feeder cells, 6000 U/ml IL-2 (Proleukin) (Peprotech) and 0,6 µg/20 ml anti-CD3 (clone OKT3) (ebioscience, Thermo Fisher). Detailed protocol was described elsewhere (43, 44), but in short; feeder cells were irradiated at 30 Gy and counted to the concentration of 20x10^6^ PBMCs/20 ml medium (X-vivo + 5% HS) to which IL-2 and anti-CD3 was added. The REP mix was then ready for the tetramer sorted MAGE-A3 positive T cells to be added. From here, the cells were given fresh IL-2 (3000 U/ml) and medium (X-vivo + 5% HS) three times a week, until the γδ T cells started growing, allowing for further analysis.

### ^51^Cr-Release Assay

Conventional ^51^Cr-release assays for cell-mediated cytotoxicity was carried out as described elsewhere (10). Briefly, target cells (cancer cells) were labeled with 100 mCi 51Cr (Perkin Elmer, Skovlunde, Denmark) in 100 µL R10 for 1 h at 37°C. After washing, the target cells were incubated with effector cells (Vγ9Vδ2 T cells) at different effector:target (E:T) ratios for 4 h at 37°C. Subsequently, the amount of radioactivity in the supernatant was measured using a gamma cell counter (Perkin Elmer Wallac Wizard 1470 Automatic gamma counter). Target cells were the cancer cell lines FM55-1 (melanoma), FM86 (melanoma), A2058 (melanoma), MDA-MB-231 (breast cancer), PC-3 (prostate cancer), U266 (myeloma) and K562 (chronic myelogenous leukemia). Also, allogeneic PBMCs (lymphocytes) from five healthy donors (HD) were thawed and rested ON and used as target cells.

Prior to cytotoxicity assays, the cancer cells or lymphocytes were left untreated or pre-treated with 10 µM ZOL for 24 hs. The rationale for pre-stimulating cancer cells with ZOL, is to make them more prone to killing by Vγ9Vδ2 T cells. ZOL inhibits farnesyl pyrophosphate synthase (FPPS), an enzyme downstream of IPP within the mevalonate pathway, which leads to accumulations off IPP intracellular making them more prone to recognition by Vγ9Vδ2 T cells. This has been shown repeatedly and the potential clinical application is being tested (45).

### xCELLigence Assay

To measure the cytotoxicity of Vγ9Vδ2 T cells against cancer cell over an extended period, the xCELLigence system was used. This assay is composed of one station with an E96 plate (xCELLigence-specific 96 well plate; ACEA biosciences, San Diego, USA), which is stored within a standard tissue culture incubator (37°C and 5% CO_2_). Its high-density electrode array, covering the bottom of E96 plates, allows this system to measure the variation in impedance throughout time. This measurement is then converted into a cell index, which can be translated into cytotoxicity.

Optimal seeding density was optimized (data not shown) and each target cell was plated out in E96 plates (10 000 cells/well for PC-3, A2058 and MDA-MD-231) and incubated for 6-24 h to promote adhesion and initial proliferation without reaching full confluency. Vγ9Vδ2 T cells were then added to each well, at a titrated 3:1, 1:1 effector-to-target ratio. The cell index was continuously measured for the next 24 h. To determine minimum impedance, 100 µl of 10% TritonX-100 (Sigma-Aldrich) was added to separate wells. Additionally, wells with Vγ9Vδ2 T cells alone were included to account for the effector cells’ contribution to the cell index. Data was analyzed with the immunotherapy module of the 185 xCELLigence RTCA Software Pro (ACEA Biosciences) as reported previously (46, 47).

**Figure 1:**
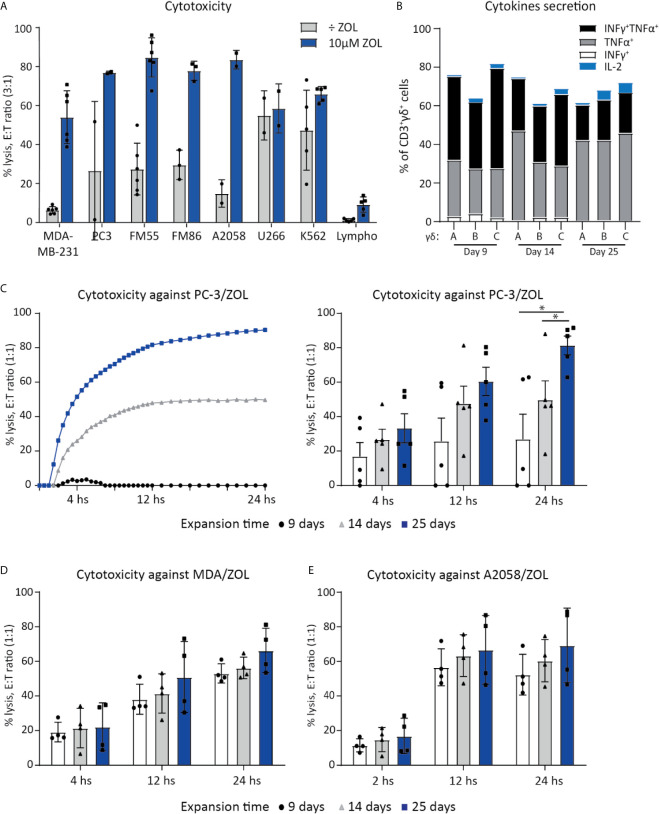
Vγ9Vδ2 T cell can efficiently kill cancer cells. **(A)** Chromium release assay (4 h) was used to test Vγ9Vδ2 T cell ability to kill cancer cell lines of various origins. Vγ9Vδ2 T cells were expanded from healthy donors (n =2-6). Effector cell (E:T) Target cell ratio (3:1). Breast cancer cell line = MDA-MB-231, Prostate cancer cell line = PC-3. Melanoma cells lines = FM55, FM86 and A2058. Hematological cancer cells lines = U266 and K562. Lymphocytes (lympho) were thawed and rested overnight prior to the assay. Vγ9Vδ2 T cell cultures used in these assays had been in culture for 14-30 days prior to the chromium release assays. Purity >90% for Vγ9Vδ2 T cell cultures was verified by flow cytometry (data not shown, see gating strategy in **Supplementary Figure 1A**). **(B)** Cytokine expression of day 9, 14 and 24 expanded Vγ9Vδ2 T cells was determined by gating on positive cells in PC-3/ZOL co-cultured with Vγ9Vδ2 T cells. Gates were set according to PC-3 co-cultured control. Three different Vγ9Vδ2 T cell cultures were analyzed, named A, B and C **(C)** Vγ9Vδ2 T cells ability to kill PC-3/ZOL cancer cell line was compared between cultures expanded for 9, 14 or 25 days, using a 24 h xCELLigence assay at effector-target cell ratio (1:1). The left graph depict one donor, showing the full 24 h xCELLigence assay, and the right graph summarizes the data from five donors (n=5 donors, repeat three times) **(D)** Comparison of percentage cytolysis at effector-target cell ratio (1:1) in xCELLigence assay, assessing Vγ9Vδ2 T cells expanded for 9, 14 or 25 days, targeting MDA-MD-231/ZOL (MDA). (n=3 donors, repeat twice) **(E)** Comparison of percentage cytolysis at effector-target cell ratio (1:1) in xCELLigence assay, assessing Vγ9Vδ2 T cells expanded for 9, 14 or 25 days, targeting A2058/ZOL (MDA). (n=3 donors, repeat twice). Statistical significance was determined by a paired T-test. *P ≤ 0.05. Error bars indicated standard error of mean (SD).

Several things were considered to rule out batch differences. All cytotox assays were conducted with Vγ9Vδ2 T cell cultures expanded from three to five different donors. Also, two different approaches were tested in cytotox assays with PC3 as a target: first the cytotox assays were conducted at the specific day of expansion, while the Vγ9Vδ2 T cells were ‘in culture’, meaning that cytotox was done on day 9, then waited 5 days to setup another cytotox on day 14, and again on day 25. The same days as the cytotox assay were performed, Vγ9Vδ2 T cells ‘in culture’ were also cryopreserved. The second approach was then to thaw cells, frozen at day 9, 14 and 25, and perform a cytotox assay in one setup. Both approaches showed similar results and representative data are shown in **Figures 1B, C**. For cytotox assay with A2058 and MDA-MB-231 as targets, only frozen Vγ9Vδ2 T cell cultures were used as effector cells.

### Antigen Cross-Presentation Assay

Vγ9Vδ2 T cells were expanded *in vitro* for 9-11 days prior to the APC assay. The setup for the APC assay was as following: *Day 1*, Vγ9Vδ2 T cells were stimulated with 1 µM ZOL and 100 U/mL IL-2. *Day 2*, Vγ9Vδ2 T cells were exposed to either a long peptide or protein and incubated 24 h to allow for cross-presentation of antigens. *Day 3*, Vγ9Vδ2 T cells were carefully washed twice with PBS to remove excess peptide or protein within the supernatant. Antigen cross-presentation was measured by IFNγ or TNFα release, using ELISPOT assay (see overview in **Figure 3**). The effector cells were either CMV-specific αβTCR T cells, or αβTCR T cells specific for gp100, MART-1 or MAGE-A3. Vγ9Vδ2 T cells and the antigen specific αβTCR T effector cells (also called ‘Teff’ in **Figures 3** and **4**), was added to the ELISPOT plate in a ratio of 4:1 (‘γδ’: ‘Teff’). This was followed by a 24 h incubation and development of the ELISPOT (see below). As a positive control, the Vγ9Vδ2 T cells were incubated with corresponding short peptide and co-cultured with the Teff. cells.

**Figure 2:**
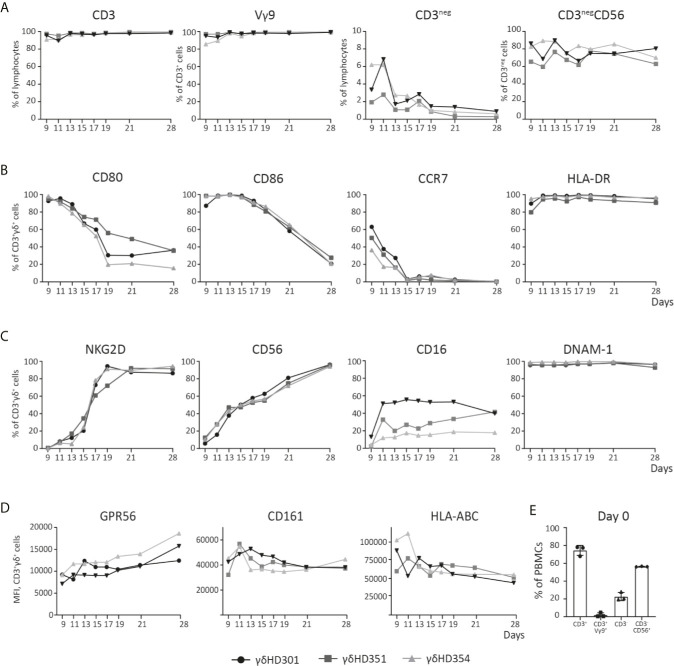
Phenotype analysis of Vγ9Vδ2 T cell cultures over time. Vγ9Vδ2 T cells cultures were expanded from three different healthy donors (HD), and analyzed by flow cytometry during culturing time *in vitro* up to 28 days. **(A)** Purity of the Vγ9Vδ2 T cell cultures is shown, by looking at both CD3_pos_ and Vγ9Vδ2 (Vγ9) T cells, but also CD3_neg_ and NK (CD3_neg_CD56) cells. **(B)** Vγ9Vδ2 T cell percentage expression of CD80, CD86, CCR7 and HLA-DR is shown. **(C)** Vγ9Vδ2 T cell percentage expression of NKG2D, CD56, CD16, and DNAM-1 is shown. **(D)** Finally, Vγ9Vδ2 T cells expression of GPR56, CD161 and HLA-ABC is shown by MFI values. **(E)** Purity analysis on day 0 of the PBMCs used for expansion to Vγ9Vδ2 T cells cultures. Complete gating strategies can be found in **Supplementary Figure 1**. (n=3).

**Figure 3:**
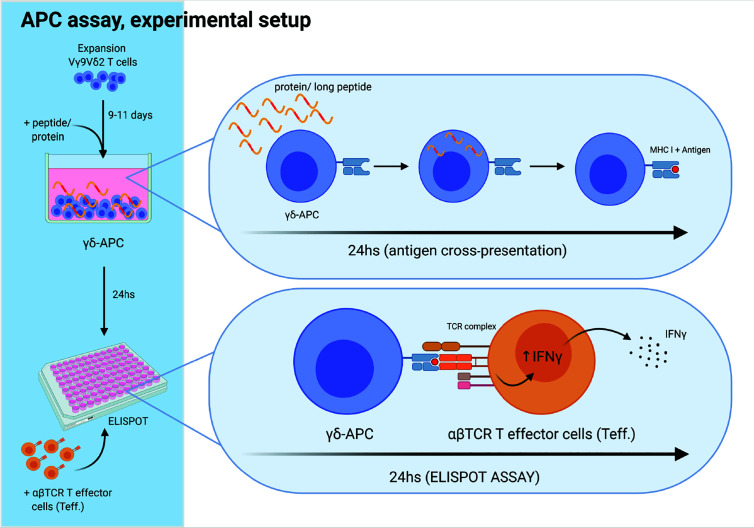
Experimental setup for antigen cross-presenting assay. The ability of Vγ9Vδ2 T cells to cross-present antigens was tested as schematically outlined. Vγ9Vδ2 T cells were expanded for 9-11 days, as described in the method section. Next, long peptide or protein was added to wells with Vγ9Vδ2 T cells, followed by 24 h incubation, to allow antigen uptake and cross-presentation. Then, the Vγ9Vδ2 T cells were washed twice to remove excess long peptide or protein. Next, Vγ9Vδ2 T cells were transferred to the ELIPOT plate and antigen specific αβTCR T effector cells (Teff) were added. IFNγ secretion following specific target recognition was measured by IFNγ ELISPOT assay. Vγ9Vδ2 T cells alone were used as negative controls. For positive controls, short peptide was added to wells containing both Vγ9Vδ2 T cells and Teff leading to maximal recognition and associated cytokine secretion by Teff.Figure is created with Biorender.

**Figure 4:**
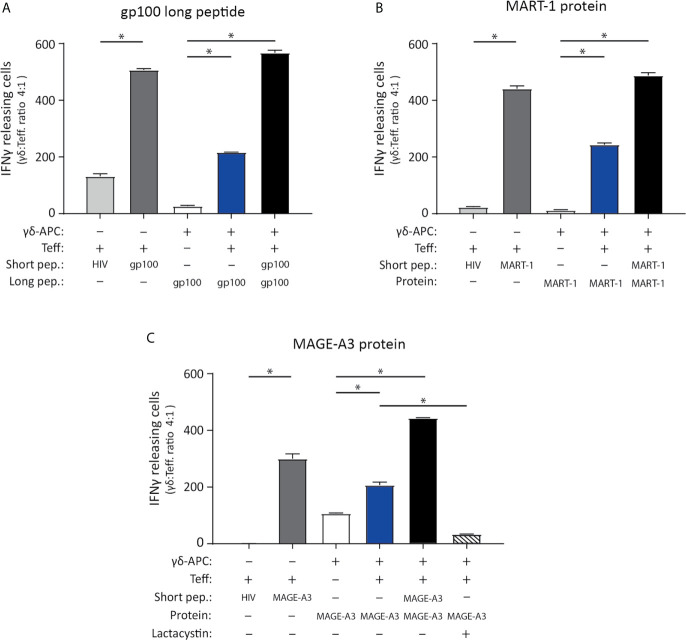
Vγ9Vδ2 T cells can cross-present tumor antigen from long tumor peptides and tumor proteins. To test the ability of Vγ9Vδ2 T cells to cross-present tumor antigen, the experimental APC setup illustrated in **Figure 3** was used. **(A–C)** Specificity of the tumor antigen specific CD8^+^ αβTCR T cells which were used as effector cells in the APC assay, is shown by ELISPOT assay. This includes three different αβTCR T effecter cells (called ‘Teff’), being specific for either gp100 (YLEPGPVTA, HLA-A*02.01-restricted), MART-1 (ELAGIGILTV, HLA-A*02.01-restricted) or MAGE-A3 peptide (EVDPIGHLY, HLA-A*01.01-restricted). Short peptides for gp100, MART-1 or MAGE-A3 peptides, were added to the ELISPOT wells as a positive control (5 nM) and confirmed specificity (dark grey bars) by comparison the unspecific short HIV peptide (ILKEPVHGV), used as negative control (light grey bars in figure a, b and c). Peptide concentration was 5 nM. (n=3, triplicates). **(A)** Tumor antigen cross-presentation was measured using IFNγ ELISPOT assay. Cross-presentation of a long gp100 peptide (29aa, 0.5 µM), recognized by gp100 specific αβTCR cells (Teff.) (n=3 triplicates). **(B)** Cross-presentation of MART-1 recombinant protein (118aa, 1.25 nM), recognized by MART-1 specific αβTCR effector cells (T.eff) (n=3 triplictes). **(C)** Cross-presentation of MAGE-A3 recombinant protein (314aa, 27 nM), recognized by MAGE-A3 specific αβTCR effector cells (Teff). Additionally, inhibition of cross-presentation of the MAGE-A3 recombinant protein was shown by addition of 50 µM lactacystin (proteasome inhibitor) (n=3, repeated in 4 independent experiments). γδ-APC refers to the Vγ9Vδ2 T cells that served as APC for antigen presentation, Teff refers to the αβTCR T effector cells, pep = peptide. All experiments were carried out in triplicates, and distribution free resampling (DFR) method was used for statistical analysis. P-value ≤ 0.05 (*) were considered statistically significant. Error bars indicated SD.

Peptide and proteins were added at the following concentrations: 0.5 µM long CMV peptide, 0.5 µM long gp100 peptide, 1.25 nM MART-1 recombinant protein and 27 nM MAGE-A3 recombinant protein. All short peptides were added as 5 nM.

### Blocking of Antigen Cross-Presentation by the Proteasome Inhibitor Lactacystin

To test if antigen cross-presentation involved the proteasome, the proteasome inhibitor Lactacystin (Sigma Aldrich) was added to Vγ9Vδ2 T cells. In short, on ‘Day 2’ of the antigen cross-presentation assay: 50µM of Lactacystin was added to the Vγ9Vδ2 T cells. After two hours of incubation at 37°C, 5% CO_2_, cells were washed twice in RPMI medium. From here, the long peptide or protein was added as described above. This means that lactacystin was added after the initial ZOL stimulation on day 1, but prior to addition of the peptide/protein on day 2. The idea was to ensure blocking of proteasome before addition of peptide/protein, to allow blocking of antigen cross-presentation.

### Peptides and Proteins Used for the APC Assay

Overview of peptides and proteins used for the APC assay can be found in **Table 2**. All peptides were obtained from KJ Ross Petersen, with a purity >70%. All proteins were obtained from Abcam.

**Table 2:** List of short peptides, long peptides and proteins used in this study.

Name	ID	HLA restriction	Size	Peptide sequence
HIV short	HIV	HLA-A*01.01	9aa	GSEELRSLY
HIV short	HIV	HLA-A*02.01	9aa	ILKEPVHGV
CMV short	CMV_nlv	HLA-A*02.01	9aa	NLVPMVATV
CMV long	CMV_480	–	40aa	VFTWPPWQAGILARNLVPMVATV QGQNLKYQEFFWDANDI
Gp100 short	Gp100_280	HLA-A*02.01	9aa	YLEPGPVTA
Gp100 long	Gp100 long	–	29aa	SRALVVTHTYLEPGPVTA QVVLQAAIPLT
MART-1 short	MART_27-36	HLA-A*02.01	10aa	ELAGIGILTV
Recombinant MART-1 protein	–	–	118aa	N- ELAGIGILTV- N
MAGE-A3 short	MAGE-A3_199-209	HLA-A*01.01	9aa	EVDPIGHLY
Recombinant MAGE-A3 protein	–	–	314aa	N- EVDPIGHLY- N

### ELISPOT Assay

ELISPOT assay was used to measure cross-presentation of antigens by Vγ9Vδ2 T cells. In brief, ELISPOT plates (nitrocellulose bottomed 96-well plates by MultiScreen MAIP N45; Millipore) were coated ON with IFNγ capture antibody (Ab) (Mabtech) and afterwards blocked by X-vivo medium. Vγ9Vδ2 T cells (target cells) were placed in the ELISPOT plate (setup in triplicates) and short control peptides were added at 5 nM (see sequence above), with and without Teff. cells. The effector cells were either CMV, gp100, MART-1 or MAGE-A3 specific αβTCR T cells. The cells were then incubated ON, after which, the plates were washed off and secondary biotinylated Ab (Mabtech) was added. After 2 h incubation, unbound secondary antibody was washed off and streptavidin conjugated alkaline phosphatase (Mabtech) was added for 1 h. Finally, unbound conjugated enzyme was washed off and the assay developed by adding BCIP/NBT substrate (Mabtech). Developed ELISPOT plates were analyzed on CTL ImmunoSpot S6 Ultimate-V analyzer using Immunospot software v5.1.

Criteria for standard protocol guidelines as well as determination of ELISPOT responses have been a challenge. In this regard, these ELISPOT assays were conducted according to the guidelines provided by CIP (48). Significance was determined by using the nonparametric distribution-free resampling (DFR) test which gives a way of formally comparing antigen-stimulated wells with negative control wells (49).

### Apoptosis Staining

To detect apoptotic and dead cells after incubation with proteasome inhibitor Lactacystin, 0.5 x 10^6^ day-9 expanded Vγ9Vδ2 T cells were seeded in a round-bottom 96-well plate in X-vivo + 5% HS. Lactacystin (Sigma Aldrich) was dissolved in sterile H_2_O and added to the wells at final concentrations of 0 µM, 1 µM, 10 µM, 25 µM, 50 µM and 100 µM. After two hours of incubation at 37°C, 5% CO_2_, cells were washed twice with PBS + 2% FBS and stained with the following extracellular antibodies in a total volume of 50 µl for 20 min at 4°C: anti-CD3 PE-Cy7, anti-TCRγ/δ FITC and anti-HLA-ABC BV711. After two washes with PBS + 2% FBS, apoptotic and dead cells were marked by staining with the Pacific Blue™ Annexin V/SYTOX™ AADvanced™ Apoptosis Kit (Invitrogen™). Annexin V binds to phosphatidylserine exposed on the outer membrane of apoptotic cells and SYTOX™ AADvanced™ Dead Cell Stain detects necrotic cells due to their loss of membrane integrity. A stain master mix was prepared by diluting Pacific Blue™ Annexin V and SYTOX™ AADvanced™ in 1X Annexin binding buffer. Cells were stained with 100 µl stain master mix per well for 30 min at 4°C and acquired on a NovoCyte Quanteon (ACEA Biosciences) without further washes.

### Statistical Analysis

Statistical analyses were conducted using Graph-Pad Prism 7 (San Diego, USA). Differences between groups were determined by a paired T test. ELISPOT responses were analyzed using distribution free resampling (DFR) method, described by Moodie et al. for statistical analysis of ELISPOT responses (49, 50). The DFR method described here was used for statistical analysis of triplicates. DFR, p ≤ 0.05 (*) were considered statistically significant. Statistical analysis was performed using Rstudio (RStudio Team (2016). RStudio: Integrated Development for R. RStudio, Inc., Boston, MA URL http://www.rstudio.com/).

## Results

### Vγ9Vδ2 T Cells Cytotoxic Capacity Increases With Culturing Time

Vγ9Vδ2 T cells ability to kill cancer cell lines of various origins, was tested in a 4 h chromium release assay. Expanded Vγ9Vδ2 T cells from 2-5 healthy donors were used as effector cells to kill target cells, with or without sensitization with ZOL (**Figure 1A**). Effective killing when sensitized with ZOL was demonstrated in three melanoma cells lines (FM55, FM86 and A2058), a prostate cancer (PC-3) and a breast cancer cell line (MDA-MB-231) reaching 60-80% lysis. Two hematological cancer cell lines, myeloma (U266) and chronic myelogenous leukemia (K562), were also efficiently killed by Vγ9Vδ2 T cells varying from 50-70% lysis, even in the absence of ZOL sensitization. To test if Vγ9Vδ2 T cells would also kill normal healthy cells, allogeneic PBMCs (called lympho), were used as target cells. The data showed that the killing of PBMCs was below 15%, even after sensitized with ZOL (5-15%) (**Figure 1A**). Thus, while Vγ9Vδ2 T cells efficiently killed cancer cells, healthy lymphocytes were mainly left untouched.

For deeper characterization of the killing capacity, we performed long-term killing over the course of 24 h using the xCELLigence assay and additionally assessed the cytotoxic abilities of Vγ9Vδ2 T cells at different time-points of expansion. Vγ9Vδ2 T cells cultured from five healthy donor, expanded for 9, 14 or 25 days, were compared in their killing potential of PC-3 sensitized with ZOL, at an effector cell (E):(T) target cell ratio of 1:1. Exemplified in **Figure 1C**, the killing of PC-3/ZOL cancer cells by Vγ9Vδ2 T cells increased with expansion time; with Vγ9Vδ2 T cells expanded for 25 days being most efficient reaching almost 100% lysis, compared to ~45% lysis and ~0-5% lysis forVγ9Vδ2 T cells expanded for 14 days or 9 days, respectively. **Figure 1C** also summarizes the data of all five Vγ9Vδ2 T cell cultures, showing a significant higher cancer cell killing capacities for cultures that were expanded the longest in culture. The enhanced killing capacity was most evident at 24 h of co-culture, with Vγ9Vδ2 T cell cultures expanded for 25 days reaching on average ~90% lysis, compared to ~50% lysis and ~30% lysis for T cells expanded for 14 days or 9 days, respectively. Importantly, when increasing the E:T ratio to 3:1, all Vγ9Vδ2 T cell cultures had comparable ability to kill prostate cancer cells (data not shown), irrespective of expansion time. In similar setups, the ability of Vγ9Vδ2 T cells to kill A2058 and MDA-MB-231, sensitized with ZOL, was tested (**Figures 1D, E**). A tendency towards a higher cancer cell killing capacities for Vγ9Vδ2 T cell cultures that were expanded the longest in culture, could also been observed here (**Figures 1D, E**). Together, all Vγ9Vδ2 T cell cultures were capable of cancer cell killing even those of short-term expansion, but cytotoxic capacity seems to increase over expansion time, with differences depending on the cells targeted.

Finally, we setup an ICS assay, to investigate the activation of Vγ9Vδ2 T cells upon tumor engagement. Vγ9Vδ2T cells were co-cultured with PC-3 with or without ZOL, for 5 hours, and expression of IFNγ, TNFα and IL-2 was measured (**Figure 1B**). Altogether, 60-80% Vγ9Vδ2 T cells expressed one or more of the cytokines upon engagement with PC-3/ZOL – this was compared to Vγ9Vδ2 T cells cocultured with PC-3 without ZOL. IL-2 expression was generally below 5% for all conditions. No significant difference was observed between 9, 14 or 25 expansion days, and hence, the difference observed in killing, could not be explained by expression of the cytokines IFNγ, TNFα and IL-2.

### Phenotype Analysis of Vγ9Vδ2 T Cell Cultures

The phenotype dynamics of Vγ9Vδ2 T cell cultures over time was analyzed by flow cytometry. Vγ9Vδ2 T cell cultures were expanded from PBMCs of three healthy donors using 10 µM ZOL and 1000 U/ml IL-2. Initially only 0.5-5% of CD3-postive cells were Vγ9Vδ2 T cells (see **Figure 2E**). After 11 days of expansion, frequency of CD3-positive T cells was above 90% for all cultures and more than 95% of these cells were Vγ9 positive). Of the remaining cells in the cultures, NK cells constituted between 2-7% at day 9-11, but continued to decline to below 2% from day 19 (**Figure 2A**). The expression of the co-stimulatory markers CD80, CD86 and CCR7 was highest in the initial expansion phase (**Figure 2B**), in particular for CCR7 where the expression by day 9 varied from ~30-60% and dropped to zero at day 15. CD80 and CD86 were more widely expressed reaching 80-100%, and a decline starting around day 13 or 15, though at a less rapid decrease. In contrast, HLA-DR expression stayed above 90% throughout the expansion period (**Figure 1C**).

It has previously been described that expanded Vγ9Vδ2 T cells can express NK cell markers involved in killing such as NKG2D (51, 52). The data in **Figure 2C** shows, that NKG2D expression increased rapidly after day 9 and reached >80% from day 19 and onwards. The expression of the adhesion molecule CD56, increased steadily with time and reached >90% expression at day 28. CD16, a molecule involved in antibody dependent cell cytotoxicity (ADCC), was also expressed but a high degree of variance (20-60%) was observed between the Vγ9Vδ2 T cell cultures – though an increase in expression from day 9 and onwards, was observed. Finally, DNAM-1 stayed above 90% throughout the expansion period (**Figure 1C**). Notably, contrary to the decreased expression of co-stimulatory markers over time, the expression of NK markers increased with time (**Figures 2B, C**).

Other markers, such as CD161 and the GPR56, has also been suggested as markers of a cytotoxic phenotype for Vγ9Vδ2 T cells. CD161 is a C-type lectin, proposed to be involved in increased IFNγ production during γδTCR activation or in response to IL-12 and IL-18 (53). GPR56 is a G protein-coupled receptor, that although still poorly defined, appears to be involved or associated with a cytotoxic phenotype of T cells (54). We found, that both CD161 and GPR56 was expressed on almost 100% of the Vγ9Vδ2 T cells from day 9-28 (see **Supplementary Figure 1D**). For transparency, the expression of both molecules is here depicted by MFI. As for GPR56 we observed an increase in MFI during culturing time, and for CD161 the opposite, a slight decrease (see **Figure 1D**). The biological relevance of these change compared to percentage expression is unknown. A similar trend was observed for HLA class I, with a decrease in expression during culturing time, a change that was difficult to observe when only looking a percentage expression (**Figure 2D** and **Supplementary Figure 1D**). The overall gating strategy for phenotype analysis of Vγ9Vδ2 T cells is shown in **Supplementary Figure 1**.

### Cross-Presentation of Virus-Specific Antigens by Vγ9Vδ2 T Cells

We next set out to test the ability of Vγ9Vδ2 T cells to cross-present tumor antigens, because Vγ9Vδ2 T cells in the early expansion (day 7-13) express co-stimulatory markers (**Figure 2A**) and previously has been shown to cross-present virus peptides (23). To verify findings by Brandes et al. (23), and to validate our assays, we first tested Vγ9Vδ2 T cells’ ability to cross-present a virus antigen. Our experimental setup is illustrated in **Figure 3**; Vγ9Vδ2 T cells were expansion for 9-11 days after which the long peptide or protein, and minimal peptide for positive control was added for 24 hs. This was followed by analysis of Vγ9Vδ2 T cells (written as ‘γδAPC’) as targets for specific αβTCR T effector cells (‘Teff.’) using ELISPOT assay. IFNγ secretion was used as a measurement for specific target recognition and hence antigen cross-presentation. In **Supplementary Figure 2A**, successful antigen cross-presentation of the CMV epitope was demonstrated in ELISPOT as the long (40aa) CMV peptide reached ~50% of IFNγ secretion compared to the positive control with short CMV peptide, which is loaded on the HLA-molecules extracellularly. This successfully supported previously data and validated the assay.

### Cross-Presentation of Tumor Antigens by Vγ9Vδ2 T Cells

The ability of Vγ9Vδ2 T cells to cross-present tumor antigens was investigated using three tumor-antigen specific CD8^+^ αβTCR T effector cells (Teff.) that recognized, gp100 or MART-1 in an HLA-A*02:01-restricted manner, or MAGE-A3 with HLA-A*01:01 restriction. These were generated by either transfection or transduction of the TCR into αβ T cells (see material and methods). The specificity of the three αβTCR T effector cells (Teff.) was established using IFNγ ELISPOT upon recognition of the minimal gp100, MART-1, or MAGE-A3 peptide (**Table 2**) compared to reactivity to a minimal HIV peptide as negative control (The grey bars of **Figures 4A–C**). Specificity of these transfected/transduced αβTCR T effector cells (Teff.) was also confirmed in chromium release assay (data not shown).

To test cross-presentation of tumor antigens by Vγ9Vδ2 T cells, expanded Vγ9Vδ2 T cells were incubated for 24 hs with; the long gp100 peptide (29aa) which contains the minimal epitope flanked on both sides by several amino acids (**Table 2**); or the recombinant MART-1 protein; or the MAGE-A3 protein. Antigen-exposed Vγ9Vδ2 T cells were used as APC (γδ-APC), and recognition by Teff. was analyzed by IFNγ ELISPOT. Specific recognition was observed for all three antigens yielding roughly 50% of the spot count in the cross-presentation situation (blue bars) compared to the peptide-loaded condition (black bar) (**Figures 4A–C**). As an additional control for Teff. potential reactivity to long peptide, we also setup an ELISPOT with only Teff., added either short HIV peptide, short gp100 peptide or long gp100 peptide. Reactivity was only observed against the short gp100 peptide (compared to the short HIV peptide), and also not against the long gp100 peptide (see **Supplementary Figure 2B**). In conclusion, our data show that Vγ9Vδ2 T cells can cross-present the tumor-associated antigens (TAA) gp100, MART-1 and MAGE-A3.

### Cross-Presentation of Tumor Antigen Is Proteasome Dependent

To further strengthen these results, we included a proteasome inhibitor, lactacystin. To ensure that lactacystin was not toxic to the cells, an apoptosis assay was performed (**Supplementary Figure 3**), confirming that addition of 50µM lactacystin did not result in significant increase of either dead or apoptotic cells, nor a decrease in living cells. Analysis of HLA class I expression in response to lactacystin, showed no significant difference in expression. For the antigen cross-presentation assay, the lactacystin was added after the initial ZOL stimulation on day 1, but prior to addition of the peptide/protein on day 2. The rationale was to ensure blocking of the proteasome before addition of long-peptide/protein, to allow blocking of antigen cross-presentation. Addition of lactacystin, in the cross-presentation assay reduced the number of IFN-γ spots significantly (bars with stripes), emphasizing that cross-presentation by Vγ9Vδ2 T cells is, at least in part, mediated by the proteasome (**Figure 4C**).

## Discussion

Vγ9Vδ2 T cells as well as αβ T cells are known to recognize infected and cancerous cells, but by very different mechanisms. αβ T cells recognize peptides bound to HLA molecules, whereas Vγ9Vδ2 T cells recognize pAg independently of HLA. As a consequence, Vγ9Vδ2 T cells recognize and kill cancer cells independently of tissue type, and its specificity is broader compared to αβ T cells at the clonal level. Most of the target molecules recognized by Vγ9Vδ2 T cells are broadly expressed on cancer cells (55). Our results support this notion by showing that Vγ9Vδ2 T cells can kill cancer cells of various histotypes, ranging from breast cancer, prostate cancer, melanoma to hematological cancers. Furthermore, the killing capacity of Vγ9Vδ2 T cells can be significantly increased upon sensitization with ZOL, with the exception of the hematological cancer cell lines, which were killed quite efficiently in the absence of sensitization.

Data from several studies have shown that Vγ9Vδ2 T cells can express both co-stimulatory and NK markers. To our knowledge, previous comparisons have merely been restricted to single time points (20, 56), stimuli dependent expression (57) or comparison of NK markers (58). Here, we aimed to observe the expression of these markers over an extended period, to obtain a better understanding of the dynamics of the phenotype over time during *in vitro* generation of Vγ9Vδ2 T cell cultures. We observed that co-stimulatory markers CD80, CD86 and CCR7 are highly expressed in the initial expansion phase, followed by a decrease, while expression of NK markers tended to increase over time. This seems to especially involve an increase in NKG2D, CD56, to a minor extent CD16 and GPR56. These markers have also been suggested by others in describing cytotoxic phenotype for Vγ9Vδ2 T cells (22, 59, 60). To investigate whether these phenotypic changes actually corresponded to changes in functionality, we compared the cytotoxic capacity of Vγ9Vδ2 T cells expanded for 9 days *versus* those expanded for 14 or 25 days. Vγ9Vδ2 T cells, independent of duration of expansion, were able to kill cancer cells. However, a significant difference in killing efficiency could be observed between the γδ T cell populations at low EC : TC ratio; with Vγ9Vδ2 T cells expanded for more than 25 days being the most efficient cancer cell killers. This was most pronounced when targeting the prostate cancer cell PC-3, whereas only a tendency could be observed when targeting MDA-MB-231 or A2058. Overall, this supports our findings that Vγ9Vδ2 T cell cultures switch from a co-stimulatory phenotype into a more effector cell type during prolonged expansion times *in vitro*.

Expression of co-stimulatory markers has traditionally been described for professional APC, such as DCs, and has been shown to be essential for priming of naïve αβ T cells (61). A comparison of antigen presenting capacity of Vγ9Vδ2 T cells with DCs has been reported elsewhere. In short, Brandes and colleagues demonstrated that *in vitro* Vγ9Vδ2 T cells are equal to DCs in their ability to present virus antigen and activate αβ T cells (20). The same study also showed that Vγ9Vδ2 T cells are capable of cross-presenting virus antigens, and as study by Capsomidis et al, showed antigen cross-presentation of a long MART-1 peptide (25 amino acids long) (22). In further consideration of tumor antigens, Himoudi et al., demonstrated that Vγ9Vδ2 T cells can cross-present long peptide as well as cancer cell derived protein, the latter requiring opsonization of target cells (24). We show cross-presentation by Vγ9Vδ2 T cells for three different tumor antigens; gp100, MART1 and MAGE-A3, as long peptide or recombinant protein and restricted by two different HLA molecules (**Figure 4**). The exact mechanism of how antigens are cross-presented is still under investigation, and cross-presentation without the involvement of the proteasome and transporter associated with antigen processing (TAP) has been described (62). However, most studies have demonstrated a mandatory requirement for proteasomal activity (25). To substantiate this notion, we took advantage of the proteasome inhibitor lactacystin which led to a highly reduced IFNγ response of MAGE-A3-specific CD8 T cells (see **Figure 4C**), strongly suggesting that the proteasome is indeed involved in cross-presentation of antigens by Vγ9Vδ2 T cells. Minor variation in efficacy can be observed between the three Teff. cells, but this has also been described elsewhere, for example by Morel et al. (63) and we still find the antigen cross-presentation to be solid for all three Teff.

Collectively, our data support the role of Vγ9Vδ2 T cells as an antigen presenting cells *in vitro*. Whether this is a physiological relevant function *in vivo* is still unknown and we do not know the exact mechanism of antigen uptake. The study of Himoudi et al., could demonstrate a significantly improved uptake of cells upon opsonisation (24). We tested uptake of protein but did not test uptake of cells in our system, but Vγ9Vδ2 T cells have been shown to take up antigen by phagocytosis (64) as well as trogocytosis (65) and it seems likely that antibody binding could possibly improve both. Based on our results, it is clear that Vγ9Vδ2 T cells can take up antigen regardless of opsonization, and cross-present the antigen to CD8 T cells.

We were able to demonstrate a shift in phenotype and cytotoxic capacity associated with culture time. However, even at early time points when the cells are highly capable of antigen cross-presentation – the cells are efficient killers. This is in agreement with previous studies showing that Vγ9Vδ2 T cells can cross-present antigens from target cells which they just had killed (24). At this stage, the Vγ9Vδ2 T cells should still express CCR7 and supposedly migrate to lymph nodes to initiate or support CD8 T cell activation. This notion is currently purely speculative, although it has been shown that Vγ9Vδ2 T cells have a supportive role for antitumor responses performed by αβ T cells (66).

Vγ9Vδ2 T cells have been tested in ACT, and the condition used for expansion of Vγ9Vδ2 T cells in the present study should be relevant for the generation of such cells for clinical application. The presented data reveal important information regarding dynamic changes during prolonged time in culture and highlight the possibility that the change in function to some extent can be monitored by changes in phenotype. Given the knowledge that *in vitro* expanded Vγ9Vδ2 T cells –do not present antigen in the absence of added tumor antigen –, the most rational setup for maximal cytotoxic effector function in ACT would be to use Vγ9Vδ2 T cells that do not express CCR7, e.g. have been expanded for 14 days or more. Alternatively, tumor antigen could be added for uptake and cross-presentation very early during the expansion, and cells be administered while they still express CCR7 on the surface, however, in that case much fewer cells. Potentially concurrent administration of IL-2 or IL15 could aid *in vivo* expansion and persistence (34, 67).

In conclusion, we show that *in vitro* expanded Vγ9Vδ2 T cells can kill cancer cells across a broad range of histotypes, cross-present tumor antigens in a proteasome-dependent manner and become more cytotoxic with culture time. We believe these dynamics of function and phenotype should be considered prior to clinical application.

## Data Availability Statement

The original contributions presented in the study are included in the article/**Supplementary Material**. Further inquiries can be directed to the corresponding author.

## Author Contributions

GHO: study design, development of methodology, data acquisition, analysis and interpretation, and writing of the manuscript. MI, AMCS, PA and SKS: data acquisition, analysis and interpretation, and revision of the manuscript. EN, RD, BM and ÖZ: development of methodology and data interpretation. PS: study supervision and design, development of methodology, data analysis and interpretation, and writing of the manuscript. All authors contributed to the article and approved the submitted version.

## Funding

The project was supported by grants from The Danish Cancer Society (R72-A4396-13-S2), The Aase and Ejnar Danielsen Foundation, The Dagmar Marshalls Foundation, the A.P. Moller foundation, The Danish Council for Independent Research (DFF – 1331 – 00095B), and Dansk Kræftforskningsfond.

## Conflict of Interest

The authors declare that the research was conducted in the absence of any commercial or financial relationships that could be construed as a potential conflict of interest.

## Abbreviations

ACT, adoptive cell therapy; APC, antigen presenting cell; HMB-PP, 4-Hydroxy-3-methyl-but-2-enyl pyrophosphate; IPP, isopentenyl pyrophosphate; ZOL, zoledronic acid.
