# Supplementary material for: Vγ9Vδ2 T Cells Concurrently Kill Cancer Cells and Cross-Present Tumor Antigens
Source: Front Immunol. 2021 Jun 2;12:645131. doi: 10.3389/fimmu.2021.645131 (PMC8208807; doi:10.3389/fimmu.2021.645131)
Supplement: Supplementary file 1 [file DataSheet_1.pdf]

SUPPLEMENTARY

FIGURE 1

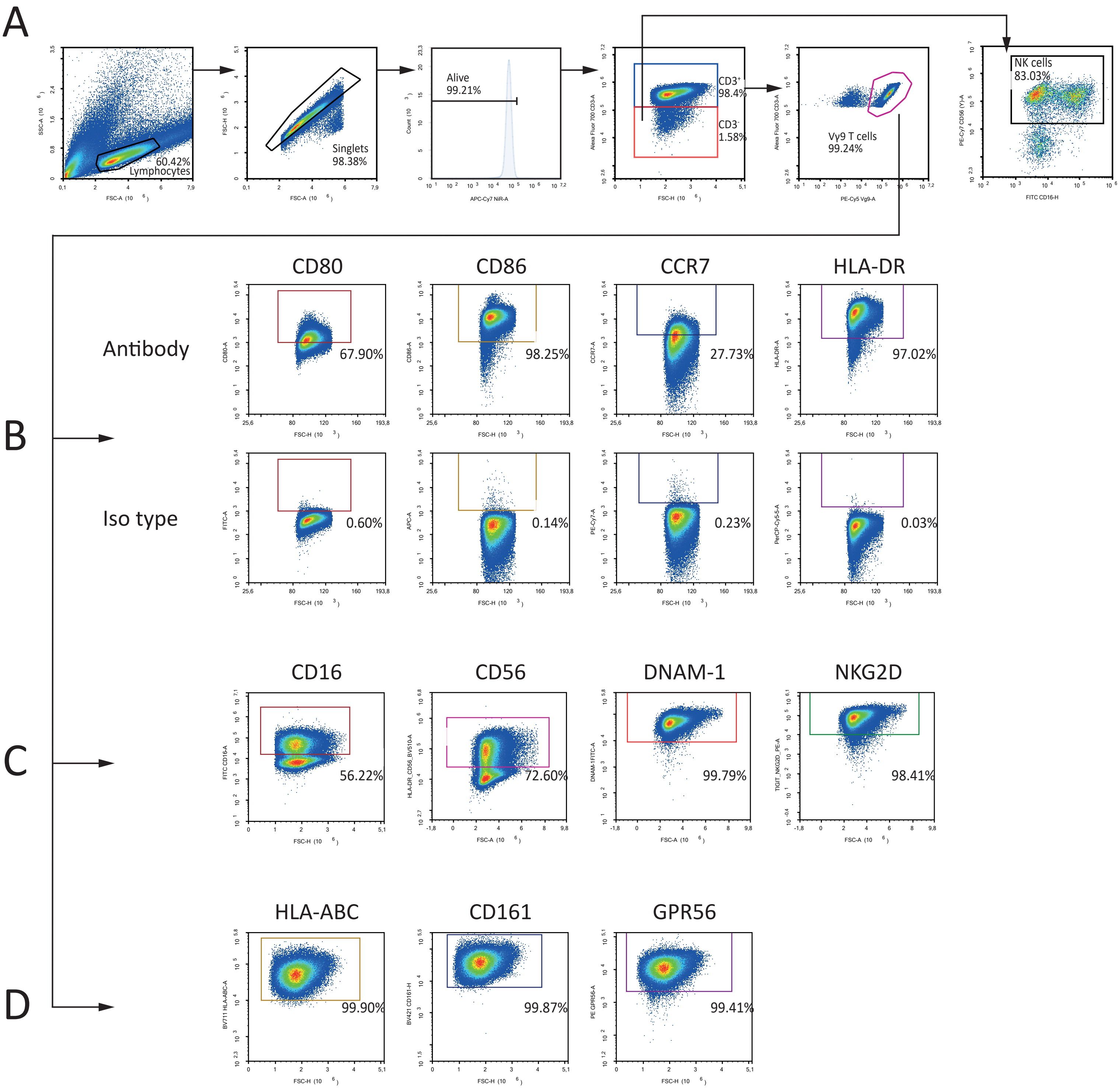

SUPPLEMENTARY FIGURE 1 | Gating strategy for purity and phenotype analysis of Vγ9Vδ2 T cell cultures. Flow analysis of Vγ9Vδ2 T cells cultures were used for purity and phenotype analysis, and a general gating strategy is shown here. **(A)** Initial gating strategy for purity analysis; First lymphocytes are gated out, then single cells and next live cells. From here cells are separated into CD3 positive, and CD3 negative cells. The CD3 positive group is then gating for Vγ9 cells - in the paper referred to as Vγ9Vδ2 T cells. The CD56 expression of the CD3 negative cells are further explored since these are the NK cells. This is the standard purity analysis used for all Vγ9Vδ2 T cell cultures in this paper. **(B)** The Vγ9Vδ2 T cells expression of CD80, CD86, CCR7 and HLA-DR, is shown here. Isotype controls for each antibody, was used to set the gates. The representative Vγ9Vδ2 T cell culture shown in this plot had been expanded for 9 days. **(C)** The Vγ9Vδ2 T cells expression of CD16, CD56, DNAM-1 and NKG2D, is shown here. NK cells were used as a positive marker to set the gates (not shown). The representative Vγ9Vδ2 T cell culture shown in this plot had been expanded for 21 days. **(D)** The Vγ9Vδ2 T cells expression of HLA-ABC, CD161 and GPR56, is shown here. The expression of each of these makers are presented with MFI values in the paper, but as depicted here, the expression of all the markers reached ~100%. The representative Vγ9Vδ2 T cell culture shown in this plot had been expanded for 21 days.

SUPPLEMENTARY

FIGURE 2

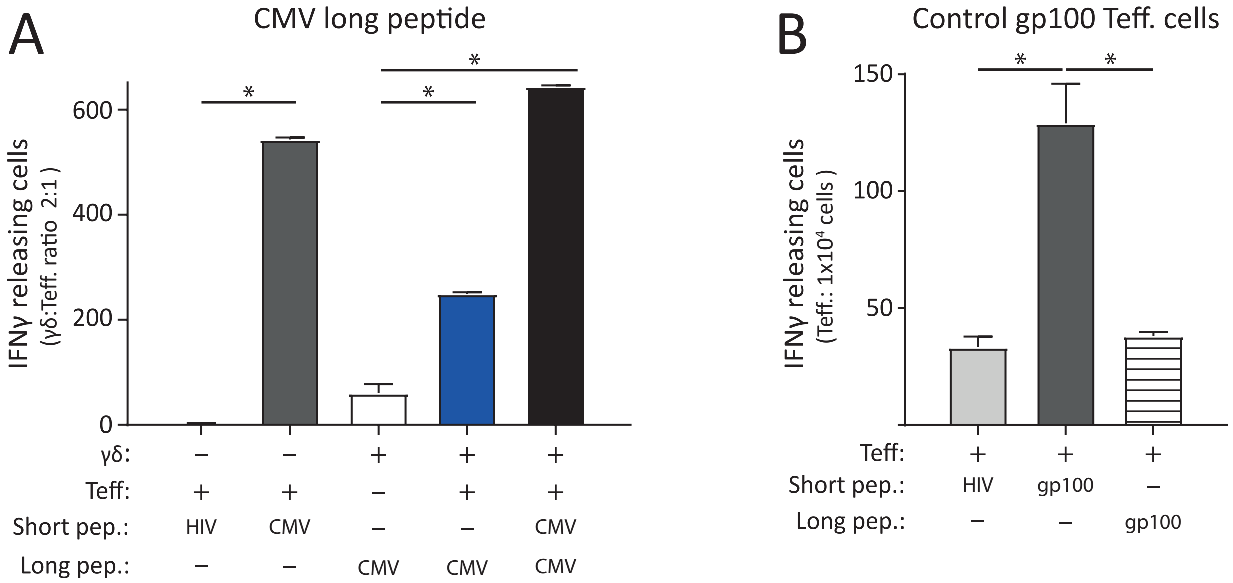

SUPPLEMENTARY FIGURE 2 | Vγ9Vδ2 T cell can cross-present virus antigen. **(A)** To test cross-presentation of the CMV virus antigen by Vγ9Vδ2 T cells, the experimental setup, as illustrated in **Figure 3**, was used. The number of IFNγ releasing cells were counted using ELISPOT assay. The specificity of the αβTCR T effector cells (Teff.) recognizing the CMV epitope (NLVPMVATV, HLA-A*02.01-restricted) was shown by comparison of an unspecific HIV peptide with the short CMV peptide (grey bars). Virus antigen cross-presentation by Vγ9Vδ2 T cells was demonstrated by yielding roughly 50% of IFNγ-spot-count in the cross-presentation situation (blue bars) compared to the positive control, where short peptide was loaded to the wells (black bar). **(B)** As an additional control for Teff. specific reactivity, an ELISPOT assay was setup with only Teff. and no γδAPC cells. The specificity of the Teff. recognizing the short gp100 epitope (YLEPGPVTA, HLA-A*02.01-restricted) in the dark grey bars was shown by comparison of an unspecific HIV peptide (light grey bars). Finally, addition of the long gp100 peptide (bars with line) show no increase in IFNγ response as compared to the unspecific HIV peptide. All experiments were carried out in triplicates, and distribution free resampling (DFR) method was used for statistical analysis. P-value ≤ 0.05 (*) were considered statistically significant. Error bars indicated SEM. CMV = cytomegalovirus, γδ = Vγ9Vδ2 T cells, Teff = αβTCR T effector cells, pep = peptide.

SUPPLEMENTARY

FIGURE 3

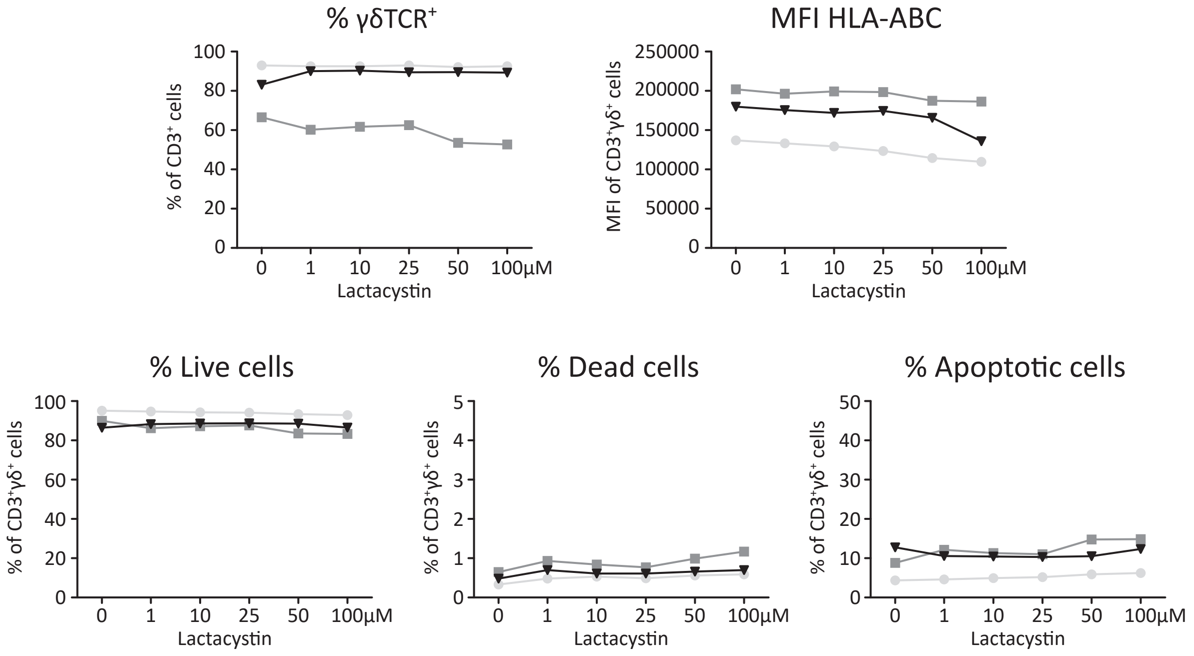

SUPPLEMENTARY FIGURE 3 | Detection of apoptotic and dead Vγ9Vδ2 T cells after incubation with the proteasome inhibitor lactacystin. Vγ9Vδ2 T cells were added lactacystin for 2 hs prior to antigen cross-presentation assay to block the proteasome. The potential toxic effect of lactacystin on Vγ9Vδ2 T cells, was investigated in this setup. The following concentration 0, 1, 10, 25, 50 and 100µM lactacystin was added to the Vγ9Vδ2 T cultures for two hours. Afterwards the viability of the Vγ9Vδ2 T cells was investigated by flow cytometry, here shown as percentage of live, dead and apoptotic cells. For this analysis the Pacific Blue™ Annexin V/SYTOX™ AADvanced™ Apoptosis Kit from Invitrogen was used. Additionally, lactacystin effect on the expression of the γδTCR and HLA-I is shown. (n=3 donors).
